# Dr. Hiram Christopher

**Published:** 1901-03

**Authors:** 


DR. HIRAM CHRISTOPHER, of St. Joseph, Mo., editor of the *Medical Herald*, died at his home January 31, 1901, aged 81 years. Dr. Christopher was a man of accomplishment and his influence in journalistic and professional circles was exercised in the direction of improvement.

